# Drug Substitution as a Potential Unintended Consequence of Antipsychotic Deprescribing in Long‐Term Care: A Retrospective Cohort Study in Ontario, Canada

**DOI:** 10.1002/alz.088510

**Published:** 2025-01-09

**Authors:** Julia G Kirkham, Daniel A Harris, Paul Nguyen, Susan E Bronskill, Colleen J Maxwell, Andrea Iaboni, Mohammad Z I Chowdhury, Dallas Seitz

**Affiliations:** ^1^ University of Calgary, Calgary, AB Canada; ^2^ Brown University, Toronto, RI USA; ^3^ ICES, Queen’s University, Kingston, ON Canada; ^4^ ICES, Toronto, ON Canada; ^5^ University of Toronto, Toronto, ON Canada; ^6^ University of Waterloo, Waterloo, ON Canada; ^7^ KITE Research Institute, Toronto Rehabilitation Institute ‐ University Health Network, Toronto, ON Canada

## Abstract

**Background:**

The overuse of antipsychotics in persons with dementia in long‐term care (LTC) has been a source of clinical concern, public attention, and policy intervention for over 30 years. Targeted quality improvement, broader awareness of risks, and other initiatives have resulted in substantial reductions in antipsychotic use in LTC settings in North America and elsewhere. Limited evidence suggests that reductions in antipsychotic use may be resulting in unintended consequences, such as substitution with alternate, but similarly harmful, psychotropic medications.

**Methods:**

We used a retrospective, matched cohort study design using linked population‐based health care databases held at ICES. LTC residents 66 years or older with dementia who were prescribed an inappropriate (i.e., without an indication aligning with the definition of appropriate antipsychotic use in LTC, including schizophrenia, Huntington’s disorder, hallucinations, delusions, or end‐of‐life care) antipsychotic medication with at least 6‐months of continuous use were identified over a 10‐year period (2008‐18). Antipsychotic users who subsequently discontinued an antipsychotic medication were matched 1:1 to persistent users on key variables and followed for up to 1‐year following antipsychotic discontinuation for new prescriptions of one or more psychotropic medications and clinical outcomes.

**Results:**

Among 26,092 LTC residents with dementia (mean age of 84 years) who were prescribed an inappropriate antipsychotic medication, 5,854 (22%) discontinued during the follow‐up period. After adjusting for key variables, new psychotropic medication prescription was not more common among antipsychotic discontinuers in the 6‐months following antipsychotic discontinuation compared to those who continued an antipsychotic medication (hazard ratio (HR) 0.90; 95% CI, 0.70‐1.15). Mortality was similar between the two groups (HR 1.01; 95% CI 0.90‐1.15) at up to 1‐year following antipsychotic discontinuation.

**Conclusion:**

Antipsychotic discontinuation in this study was not associated with medication substitution. These results supports other studies indicating that antipsychotic discontinuation in dementia can be safe but has questionable effect on mortality. While medication use trends in LTC have shown increases in other psychotropic medication use alongside antipsychotic reductions, this study suggests that this may be driven by factors other than substitution, such as increasing complexity of LTC residents, including higher prevalence of mental health disorders other than dementia.

